# Nitrogenase Activity in Thermophilic Chemolithoautotrophic Bacteria in the Phylum *Aquificae* Isolated under Nitrogen-Fixing Conditions from Nakabusa Hot Springs

**DOI:** 10.1264/jsme2.ME18041

**Published:** 2018-11-23

**Authors:** Arisa Nishihara, Katsumi Matsuura, Marcus Tank, Shawn E. McGlynn, Vera Thiel, Shin Haruta

**Affiliations:** 1 Department of Biological Sciences, Tokyo Metropolitan University Minami-Osawa, Hachioji, Tokyo 192–0397 Japan; 2 Earth-Life Science Institute, Tokyo Institute of Technology Ookayama, Meguro-ku, Tokyo 152–8551 Japan; 3 Biofunctional Catalyst Research Team, RIKEN Center for Sustainable Resource Science Wako-shi 351–0198 Japan; 4 Blue Marble Space Institute of Science Seattle, WA 98145–1561 USA

**Keywords:** nitrogen fixation, thermophile, *Aquificales*, *nifH* gene, hydrogen-oxidizing bacteria

## Abstract

The phylum *Aquificae* comprises chemolithoautotrophic thermophilic to hyperthermophilic bacteria, in which the nitrogenase reductase gene (*nifH*) has been reported. However, nitrogen-fixing activity has not yet been demonstrated in members of this deeply branching bacterial phylum. We isolated two thermophilic diazotrophic strains from chemosynthetic microbial communities in slightly alkaline hot springs (≥70°C) in Nakabusa, Nagano Prefecture, Japan. A phylogenetic analysis based on 16S rRNA genes identified these strains as members of the genus *Hydrogenobacter* within *Aquificae*. Their NifH sequences showed 96.5 and 97.4% amino acid sequence identities to that from *Hydrogenobacter thermophilus* TK-6. Nitrogenase activity, measured by acetylene reduction, was confirmed in both strains at 70°C. These novel strains grew under semi-aerobic conditions by using CO_2_ as the sole carbon source and N_2_ as the sole nitrogen source in media containing hydrogen and/or thiosulfate. To the best of our knowledge, this is the first demonstration of active nitrogen fixation in thermophilic bacteria at 70°C and in the phylum *Aquificae*.

The phylum *Aquificae* is a deeply branching bacterial phylum that contains chemolithoautotrophic thermophilic to hyperthermophilic bacteria. The phylum *Aquificae* consists of a single order, *Aquificales*, and three families, *Aquificaceae*, *Hydrogenothermaceae*, and *Desulfurobacteriaceae*, as well as *Thermosulfidibacter takaii*, a member of uncertain taxonomic affiliation within the phylum (10, 31). The complete genome sequences of nine *Aquificae* are available in the public database, and two of them, *Hydrogenobacter thermophilus* TK-6^T^ (WP_012963773) and *Thermocrinis albus* DSM 14484^T^ (WP_012991466) contain *nifH* genes encoding nitrogenase reductase, a key enzyme for biological nitrogen fixation (45, 48). Environmental putative *nifH* gene sequences that phylogenetically cluster with *nifH* sequences from isolated *Aquificae* strains have been obtained from various (hyper)thermophilic microbial communities (7, 8, 13, 22, 29). However, nitrogen-fixing activity has not been demonstrated in any members of the phylum *Aquificae*.

At Nakabusa hot springs in Japan, chemosynthetic microbial communities develop in slightly alkaline sulfidic hot springs with temperatures higher than 70°C, similar to many other thermophilic terrestrial hot springs worldwide (24–28, 34, 35, 40, 43, 44, 46). In these communities, *Aquificae* are the dominant bacteria and are assumed to fix carbon and provide organic compounds as primary producers to the communities (2, 16). Despite low concentrations of nitrogen compounds, *e.g.*, ≤6.1 μmol L^−1^ of ammonium in the alkaline hot spring water at Nakabusa, high biomass production levels have been observed in chemosynthetic microbial communities, and have sometimes been higher than those achieved by photosynthetic productivity (12, 16).

We previously reported nitrogen fixation activity at 70°C in chemosynthetic communities from Nakabusa hot springs, which was related to autotrophic sulfate-reducing metabolism (28). Subsequent molecular analyses targeting *nifH* revealed a high abundance of *Aquificae*-related putative *nifH* sequences, indicating members of *Aquificae* as potentially dominant diazotrophs in these communities (29).

In the present study, we isolated two thermophilic diazotrophic *Aquificae* from the chemosynthetic microbial communities in Nakabusa and demonstrated their nitrogen-fixing abilities at 70°C.

## Materials and Methods

### Sample collection

Microbial mats (pale-tan color) and streamers (pale-tan and gray color) were collected on March 29 and May 7, 2017 at [36°23′20″N 137°44′52″E] (Wall Site) and [36°23′33″N, 137°44′52″E] (Stream Site), respectively, in Nakabusa hot springs, Japan. This hot spring water is slightly alkaline (pH 8.0 to 8.9) and contains sulfide (0.10 to 0.25 mmol L^−1^) and small amounts of nitrogen compounds (5.0 to 6.1 μmol L^−1^ of ammonium, and below detection limits of nitrate and nitrite), as previously described (12, 15, 26, 27). The samples collected in 30-mL test-tubes filled with hot spring water were brought to the laboratory, stored at room temperature for up to 12 d, and used for bacterial cultivation at 70°C within 12 d.

### Cultivation of nitrogen-fixing bacteria

Nitrogen compound-free (N-free) modified TK-6 medium (10, 11) was prepared by replacing (NH_4_)_2_SO_4_ with Na_2_S_2_O_3_ (1 g L^−1^). CO_2_ gas was used as the sole carbon source in the medium. A total of 0.75 mL of a modified trace mineral solution was used per L medium (10), and the modified trace mineral solution was supplemented with 0.5 g Na_2_-EDTA·2H_2_O and 0.1 g Na_2_SeO_4_ (L^−1^), while nitrilotriacetic acid, H_2_SeO_3_, and KAl (SO_4_)_2_·12H_2_O were omitted. The pH of the medium was adjusted to 7.0 using HCl prior to autoclaving. Twenty milliliters of the medium was placed into a 120-mL glass vial. The vial was sealed with a butyl rubber stopper and aluminum cap, and autoclaved after the gas phase was replaced with N_2_:CO_2_ (4:1, v:v). After autoclaving, 15–30% vol. H_2_ gas and 1–10% vol. O_2_ gas were aseptically added to the gas phase (described in detail below).

### Enrichment of nitrogen-fixing thermophilic bacteria

Microbial mats and streamers were homogenized using an aseptic glass homogenizer. Two hundred microliters of the homogenized samples were inoculated into 20 mL of N-free modified TK-6 medium and incubated at 70°C under static conditions in the presence of 30% vol. of H_2_ gas. One milliliter of each culture was subcultured 4 times every 1–2 weeks into fresh N-free modified TK-6 medium for the enrichment of nitrogen-fixing bacteria under two conditions of 1 and 5% vol. O_2_ as well as 30% vol. of H_2_ gas in the headspace of the vials.

### Isolation of nitrogen-fixing thermophilic bacteria

**Fig. 1:**
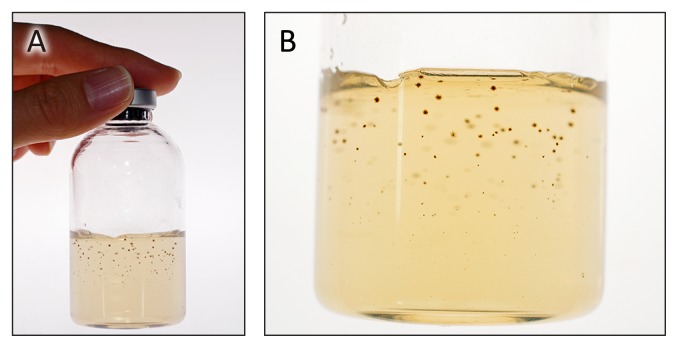
Photo images showing colony formation in a solidified medium. These images showed isolated strain 1–6 growing in a medium solidified with gellan gum. A, Dark-colored colonies formed in 30 mL of solidified medium in a 70-mL vial; B, Magnified portion of the vial.

Solid medium of N-free modified TK-6 medium containing 0.8% (w/v) gellan gum (Wako, Osaka, Japan) was prepared in sealed vials as described above. A diluted enrichment culture was injected into the vials before cooling and then solidified. Different O_2_ concentrations (1, 5, 8, or 10% vol.) in the gas phase were applied for appropriate conditions to obtain visible colonies (Fig. 1) for isolation into an axenic culture. Visible colonies were picked up aerobically and repetitively subcultured at least three times in fresh medium until an axenic culture was achieved. Purity of the isolates was confirmed by phase-contrast microscopy observations and 16S rRNA gene sequencing.

### Testing the requirement for dinitrogen and oxygen for growth

Bacterial isolates were pre-cultured in N-free modified TK-6 medium in 70-mL vials. The gas phase consisted of N_2_:CO_2_:H_2_ (4:1:2, v:v:v) with 5% vol. O_2_. One hundred microliters of the pre-culture solutions in the stationary phase of growth ([2.9 to 3.9]×10^7^ cells mL^−1^) were inoculated into 20 mL of N-free modified TK-6 medium in 70-mL vials under a gas phase of either N_2_:CO_2_:H_2_ (4:1:2, v:v:v) or Ar:CO_2_:H_2_ (4:1:2, v:v:v) with 1, 5, or 10% vol. O_2_. Vials were incubated at 70°C and shaken by hand once a day for 5 s. When indicated, 2 mmol L^−1^ of NH_4_Cl was added to the medium. Bacterial growth was assessed by cell counting using a phase-contrast microscope (AXIO Imager A2; Carl Zeiss, Oberkochen, Germany) and a counting chamber (SLGC, Saitama, Japan). The experiment was conducted in triplicate.

### DNA extraction and phylogenetic analysis based on 16S rRNA and *nifH* genes

DNA was isolated from bacterial cells following a combined protocol of mechanical disruption (bead beating) and chloroform phenol extraction, as described by Noll *et al.* (30). The 16S rRNA genes and putative *nifH* genes of bacterial isolates were amplified using the 16S rRNA gene primers 27F2/1492R2 (20, 21) and *nifH* gene primers PolF/PolR (33) under the standard PCR conditions given in the respective references. PCR was performed using *ExTaq* polymerase (Takara, Kusatsu, Japan) as described previously (28, 29). Purified PCR products were prepared and sequenced using BigDye terminator kit v3.1 on an ABI 3130 Genetic Analyzer (Applied Biosystems, Foster city, CA, USA) according to the standard protocol. *nifH* gene sequences were translated into amino acid sequences using the standard code in MEGA7 (19). The deduced NifH sequences were confirmed to contain one required residue, Cys 97 (protein numbering for NifH in *Azotobacter vinelandii*; PCR products were of insufficient lengths to contain both Cys 97 and Cys 132), which is a 4Fe-4S iron sulfur cluster ligating cysteine, and were used to construct a phylogenetic tree as previously reported (9, 29). NifH and 16S rRNA gene sequences were aligned in ClustalW with default settings implemented in MEGA7 (19). Phylogenetic trees for the 16S rRNA gene and NifH sequences were reconstructed using the Maximum Likelihood method with the Tamura-Nei model in MEGA7 (19) and the WAG model in the ARB program package (23), respectively. The robustness of the tree topologies was tested with 500 (for 16S rRNA) or 100 (for NifH) bootstrap replicates.

### Acetylene reduction assay

Nitrogenase activity was measured using the acetylene reduction method (1, 39). The production of ethylene from acetylene was quantified using a GC-2014 gas chromatograph (Shimadzu, Kyoto, Japan) equipped with an 80/100 Porapak T (GL Science, Tokyo, Japan) column. Three hundred microliters of the headspace gas of each bacterial culture vial was injected once into the column. A flame ionization detector was used with nitrogen as the carrier gas. The run conditions of the injection and detection port temperatures were 100°C and 50°C, respectively. Ethylene production was calculated by a standard curve generated from standard ethylene gas (GL Science).

### Sample preparation for the acetylene reduction assay

The nitrogen-fixing activities of the isolates in solid media were tested as follows: 300 μL of culture solutions were injected into 8 mL of N-free modified TK-6 medium containing 0.8% gellan gum and N_2_:CO_2_ (4:1, v:v) gas in the headspace of 20-mL sealed vials. H_2_ (~25% vol.) and O_2_ (1 to 10% vol.) were added to the headspace prior to the inoculation. After incubation at room temperature overnight to solidify the medium, vials were incubated at 70°C for 3 d with the addition of 15% vol. of 99.99% acetylene gas (Tatsuoka, Chiba, Japan). This experiment was conducted in duplicate.

To assess acetylene reduction in liquid medium, bacteria were cultivated for 2 d in N-free modified TK-6 medium under N_2_:H_2_:CO_2_ (5:4:1, v:v:v) and the addition of 5% vol. O_2_. Two milliliters of the cultures were placed into a 7-mL sealed vial. The headspace was replaced with Ar:H_2_:CO_2_ (5:4:1, v:v:v). NH_4_Cl (2 mmol L^−1^) was added when indicated.

After 10-min pre-incubation at 70°C in a water bath, 15% vol. of 99.99% acetylene gas (Tatsuoka) was injected into the headspace. After 3-h incubation at 70°C, 200 μL of 37% formaldehyde was added to stop the reaction producing ethylene. Examination under each condition was conducted in triplicate.

A negative control without bacteria was prepared to estimate any ethylene background generated from non-biological sources.

### Influence of molybdate on acetylene reduction

Bacteria were cultivated for 2 d in liquid N-free modified TK-6 medium under the N_2_:H_2_:CO_2_ (5:4:1, v:v:v) or N_2_:CO_2_ (4:1, v:v) gas phase with the addition of 5% vol. O_2_. Two milliliters of each culture was placed into a 7-mL sealed vial. The headspace was replaced with Ar:H_2_:CO_2_ (5:4:1, v:v:v) with O_2_ (5% vol.) or Ar:CO_2_ (9:1, v:v), respectively. A total of 20 mmol L^−1^ of sodium molybdate was added when indicated. Sample preparation for the acetylene reduction assay was the same as that described above.

### Nucleotide sequence accession numbers

16S rRNA and *nifH* gene sequences were deposited in DDBJ/EMBL/GenBank with the accession numbers LC375847 (strain 1–6) and LC375848 (strain 2–18) for the 16S rRNA gene and LC375850 (strain 1–6) and LC375849 (strain 2–18) for the *nifH* gene.

## Results

### Isolation of aerobic nitrogen-fixing bacteria

After homogenization, pale-tan microbial mats and two types of streamers (pale-tan and grayish) were cultivated under diazotrophic conditions in a N_2_:CO_2_:H_2_:O_2_ atmosphere. After repetitive cultivation, bacterial growth was observed in all cultures containing both 1% and 5% vol. O_2_ in the headspace. The cultures were transferred into solid media under 1 to 10% vol. O_2_ and visible colonies were obtained in all cultures. Twenty-seven colonies were selected, purified, and used in the acetylene reduction assay tests: 14, 11, 1, and 1 isolates were obtained from the cultures with 1, 5, 8, and 10% vol. O_2_, respectively (Fig. 1). In seven out of the 27 isolated samples, ethylene was clearly produced in acetylene reduction assays under a N_2_:CO_2_:H_2_:O_2_ atmosphere (data not shown), indicating that these seven isolates exhibited nitrogen-fixing abilities under the conditions tested. Based on 16S rRNA gene sequence analysis, the seven isolates were classified into two phylotypes; six sequences showed 100% nucleotide sequence identity to one another. The 16S rRNA gene sequences of these six strains were found in all samples; pale-tan microbial mats (four isolates), pale-tan streamers (one isolate), and gray streamers (two isolates). The representative isolate, strain 1–6, obtained from pale-tan mats with 5% vol. O_2_ in the vial headspace, was used for further analyses. Strain 2–18 was isolated from a gray streamer at 5% vol. O_2_.

**Fig. 2:**
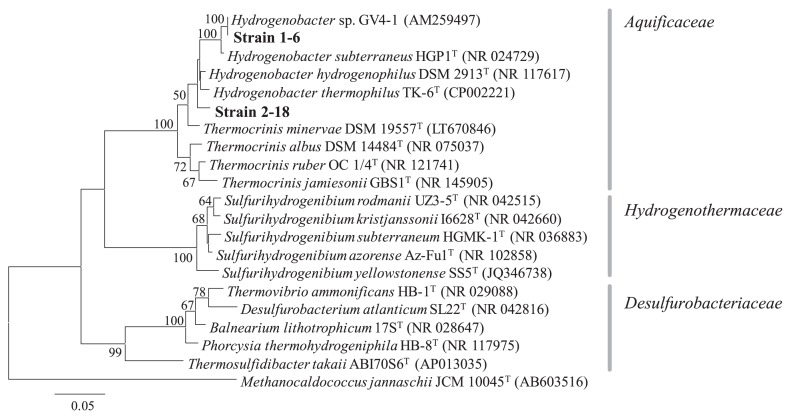
A molecular phylogenetic tree by the maximum-likelihood method based on 16S rRNA gene sequences of isolated strains 1–6 and 2–18 (shown in bold), with close relatives in the phylum *Aquificae*. Bootstrap values higher than 50 are shown. The scale bar represents a substitution rate of 5 nucleotides per 100 nucleotides.

The results of the phylogenetic analysis of 16S rRNA genes indicated that both isolates were closely related to species of the genera *Hydrogenobacter* and *Thermocrinis* (Fig. 2). A BLAST search supported this affiliation, with the closest cultivated relatives of strain 1–6 being *Hydrogenobacter* sp. GV4-1 (4) with 99.9% identity and *H. subterraneus* HGP1^T^ (42) with 98.7% identity, and with the closest cultivated relative of strain 2–18 being *H. hydrogenophilus* DSM 2913^T^ (6, 18) with 97.6% identity. The two isolates shared 95.3% 16S rRNA gene nucleotide identity with each other, indicating that they are representatives of two different species within the genus *Hydrogenobacter*.

### *nifH* sequences of isolates

**Fig. 3:**
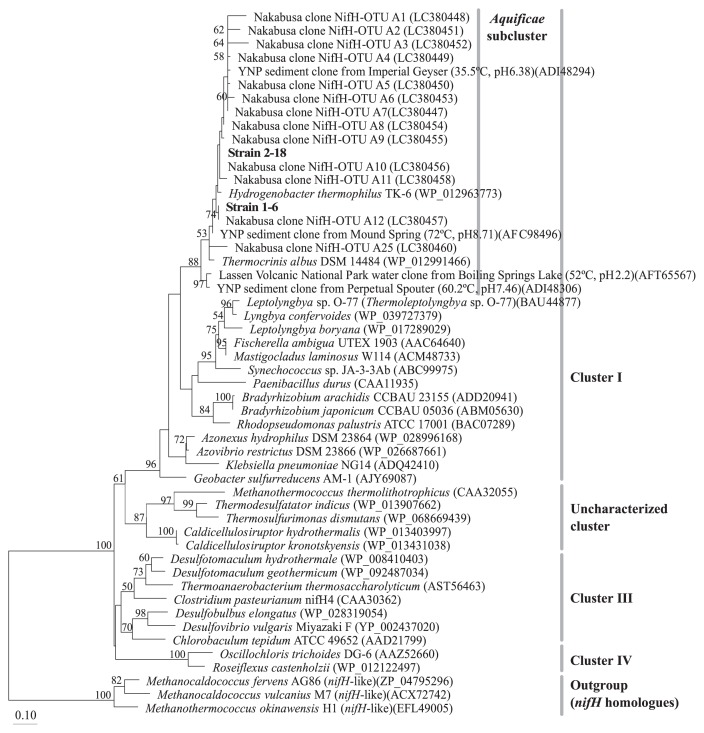
Positions of NifH amino acid sequences of strains 1–6 and 2–18 (shown in bold) and closely related sequences in a maximum-likelihood inferred phylogenetic tree. YNP, Yellowstone National Park. Bootstrap values higher than 50 are shown. The scale bar represents 10 amino acid substitutions per 100 amino acids. Environmental clones from Nakabusa hot springs (72–77°C, pH 8.5–8.9) are shown in the *Aquificae* subcluster (29). NifH clusters defined by Zehr *et al.* are also shown (47).

Putative *nifH* fragments were successfully amplified by PCR from the seven isolates and their DNA sequences were elucidated. The *nifH* gene sequences from the isolates were classified into two phylotypes showing the same relationship as that based on 16S rRNA gene sequences. As shown in Fig. 3, the deduced NifH sequences of strains 1–6 and 2–18 clustered with *H. thermophilus* TK-6^T^ and *T. albus* DSM 14484^T^, as well as environmental clones from microbial mats and streamers from Nakabusa (29), from hot spring sediments in Yellowstone National Park, and from Boiling Springs Lake water in Lassen Volcanic National Park, USA (7, 8, 22, 36). The NifH sequences of strains 1–6 and 2–18 showed 96.5% and 97.4% amino acid sequence identities, respectively, to that of *H. thermophilus* TK-6^T^ (WP_012963773). The two isolates shared 96.6% NifH sequence identity with each other.

### Growth under nitrogen-fixing conditions and acetylene reduction activity of isolates

**Fig. 4:**
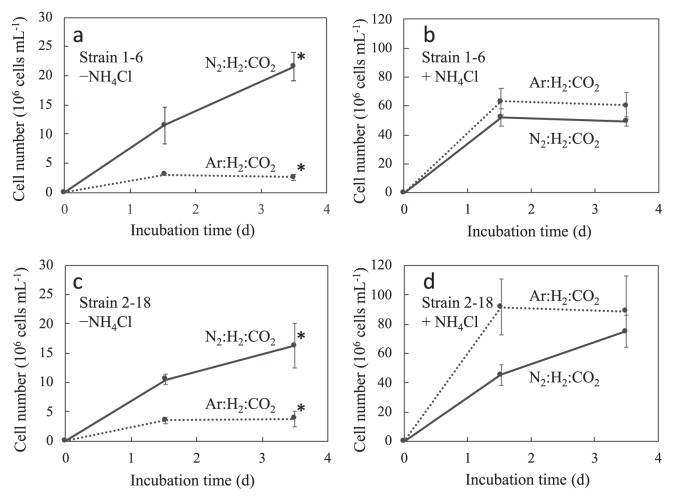
Growth of strains 1–6 (a) (b) and 2–18 (c) (d) under N_2_ and argon atmospheres. Strains 1–6 and 2–18 were cultivated without NH_4_Cl (a) (c) or with 2 mmol L^−1^ of NH_4_Cl (b) (d) under the gas phase of N_2_:H_2_:CO_2_ (5:4:1) or Ar:H_2_:CO_2_ (5:4:1) with 5% oxygen. Cell numbers in the culture solution were counted under a microscope using a counting chamber (SLGC). Error bars represent the standard deviation of three replicates. An asterisk represents samples with significant differences (Student’s *t*-test, *P*<0.05) between bacterial growths on day 3.5 under the gas phase of N_2_:H_2_:CO_2_ (5:4:1) or Ar:H_2_:CO_2_ (5:4:1) with 5% oxygen.

Diazotrophic growth was examined to confirm the nitrogen-fixing abilities of strains 1–6 and 2–18. These strains were cultivated in nitrogen-free liquid medium under Ar:H_2_:CO_2_ or N_2_:H_2_:CO_2_ with 5% vol. of O_2_ (Fig. 4a and c). Both strains showed a small increase in cell numbers after 1.5 d of cultivation under nitrogen-free conditions (argon atmosphere, Fig. 4a and c), and this may have been due to the small amounts of nitrogen compounds derived from the pre-culture; however, no further growth was observed. In contrast, under the N_2_ gas atmosphere, cell numbers continuously increased and reached 21.5±1.21×10^6^ cells mL^−1^ for strain 1–6 and 14.6±3.44×10^6^ cells mL^−1^ for strain 2–18 after 3.5 d of cultivation (Fig. 4a and c). The cell densities of strains 1–6 and 2–18 were 9.2- and 4.2-fold higher, respectively, under the nitrogen atmosphere than under the argon atmosphere. No marked differences in final cell densities (after 3.5 d of cultivation) were observed in the presence or absence of N_2_ in medium containing NH_4_Cl (*P* values >0.05) (Fig. 4b and d).

**Fig. 5:**
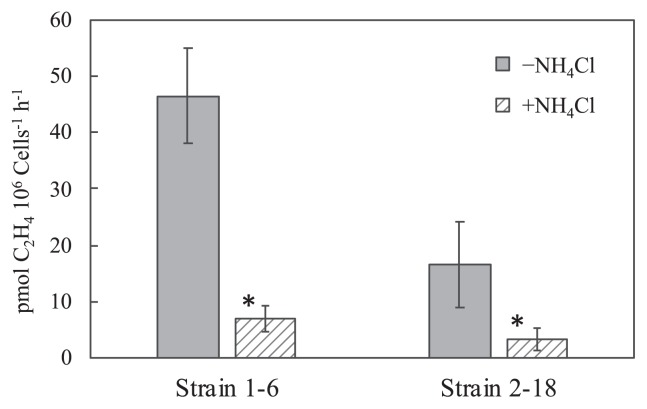
Acetylene reduction activities of strains 1–6 and 2–18 in the absence and presence of NH_4_Cl. Strains 1–6 and 2–18 were cultivated in N-free modified TK-6 medium under the gas phase of N_2_:H_2_:CO_2_ (5:4:1) with 5% oxygen for 2 d. Ethylene production was measured under Ar:H_2_:CO_2_ (5:4:1) with 5% vol. oxygen after a 3-h incubation at 70°C. Error bars represent the standard deviation of three replicates. An asterisk represents samples with significant differences (Student’s *t*-test, *P*<0.05) between without NH_4_Cl (−NH_4_Cl) and with NH_4_Cl (+NH_4_Cl) in the 3-h incubation for the measurement of ethylene production.

The nitrogenase activities of both strains were observed at 70°C, as shown in Fig. 5; 46.5±8.54 pmol C_2_H_4_×10^6^ cells^−1^ h^−1^ in strain 1–6 and 16.6±7.51 pmol C_2_H_4_×10^6^ cells^−1^ h^−1^ in strain 2–18 in nitrogen-free medium with 5% vol. of O_2_. Nitrogenase activity in the presence of 2 mmol L^−1^ NH_4_Cl in the acetylene reduction assay was observed in both isolates, but was lower than that in the absence of NH_4_Cl (*P* values <0.05), showing 7.01±2.23 pmol C_2_H_4_×10^6^ cells^−1^ h^−1^ in strain 1–6 and 3.37±2.10 pmol C_2_H_4_×10^6^ cells^−1^ h^−1^ in strain 2–18.

### Dependence of growth on oxygen concentrations

**Fig. 6:**
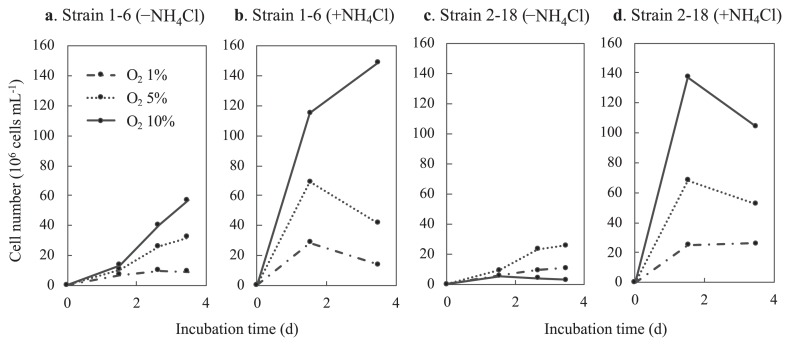
Growth of strains 1–6 (a) (b) and 2–18 (c) (d) at different O_2_ concentrations. Strains 1–6 and 2–18 were cultivated without NH_4_Cl (a) (c) or with 2 mmol L^−1^ of NH_4_Cl (b) (d) under the gas phase of N_2_:H_2_:CO_2_ (5:4:1) with 1, 5, and 10% vol. oxygen. Cell numbers in the culture suspension were counted under a microscope. Only one representative replicate (of three) are shown for each condition.

To examine the optimal O_2_ concentrations for growth under nitrogen-fixing conditions, growth under different initial concentrations of O_2_ was compared in the presence of both thiosulfate and H_2_. As shown in Fig. 6a, strain 1–6 showed faster growth at 10% vol. O_2_ than at 1 and 5% vol. O_2_; however, initial growth for 1.5 d was similar. In contrast, in Fig. 6c for strain 2–18, growth was not observed at 10% vol. O_2_, but was better at 5% vol. O_2_ than at 1% vol. O_2_. In the presence of NH_4_Cl, growth at 10% O_2_ was better than under the other conditions for both strains (Fig. 6b and d). The semi-aerobic growth of both strains was also observed not only under conditions containing both thiosulfate and hydrogen, but also under conditions in which thiosulfate or hydrogen was the sole electron donor (data not shown).

### Effects of molybdate on nitrogenase activity

**Fig. 7:**
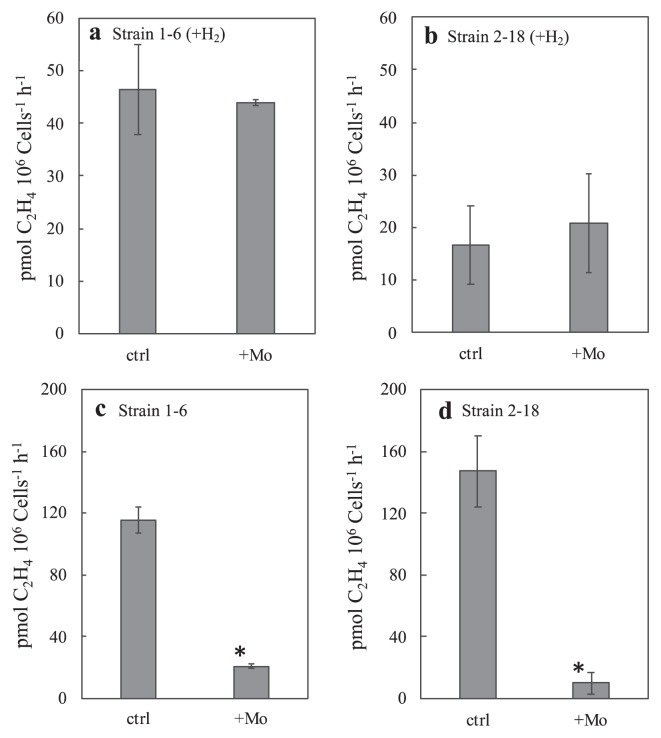
Acetylene reduction activities of strains 1–6 (a) (c) and 2–18 (b) (d) in the presence and absence of molybdate (+Mo). The column for the control (“ctrl”) shows the same data as that shown for “−NH_4_Cl” in Fig. 4. Strains 1–6 and 2–18 were cultivated in N-free modified TK-6 medium containing thiosulfate under the gas phase of N_2_:H_2_:CO_2_ (5:4:1) for (a) (b) or N_2_: CO_2_ (4:1) for (c) (d) with 5% vol. oxygen. Ethylene production by cultures was measured under Ar:H_2_:CO_2_ (5:4:1) with 5% vol. oxygen (a) (b) or Ar:CO_2_ (9:1) without oxygen (c) (d) during a 3-h incubation at 70°C. Error bars represent the standard deviation of three replicates. An asterisk represents samples with significant differences (Student’s *t*-test, *P*<0.05) between without molybdate (ctrl) and with molybdate (+Mo) in a 3-h incubation for the measurement of ethylene production.

We previously reported that the anaerobic nitrogenase activities of chemosynthetic thermophilic microbial mats and streamers in Nakabusa were mostly suppressed by molybdate (28), an inhibitor of dissimilatory sulfate reduction and other reactions involving sulfate adenylyltransferase (ATP sulfurylase) (32). The effects of molybdate on the activities of strain 1–6 and 2–18 were examined using 2-d pre-cultivated cultures under 5% vol. O_2_ (Fig. 7). The acetylene reduction activities of these strains in the presence of both thiosulfate and H_2_ under 5% vol. O_2_ were not inhibited by the addition of molybdate at a final concentration of 20 mmol L^−1^ (*P* values >0.05) (Fig. 7a and b). On the other hand, these activities in the absence of H_2_ and O_2_ during activity measurements for 3 h were markedly inhibited by molybdate in both strains in cells that had been pre-cultivated with thiosulfate and 5% vol. O_2_ without H_2_ (*P* values <0.05) (Fig. 7c and d).

## Discussion

In the present study, seven strains and two distinct phylotypes of aerobic thermophilic chemolithoautotrophic nitrogen-fixing bacteria in the phylum *Aquificae* were isolated from Nakabusa hot springs, Japan. The N_2_-fixing capability of two novel strains in the phylum *Aquificae* was demonstrated by positive acetylene reduction activities and diazotrophic growth. Genome analyses indicated that two species of bacteria in *Aquificae*, *H. thermophilus* TK-6^T^ (WP_012963773) and *T. albus* DSM 14484^T^ (WP_012991466), possessed putative *nifH* genes, whereas their active nitrogen-fixing abilities have never been demonstrated. Kawasumi *et al.* reported that *H. thermophilus* TK-6 did not grow using nitrogen gas as a sole nitrogen source (14). Consistent with these findings, we found that *H. thermophilus* TK-6 (kindly provided by Dr. H. Arai) did not exhibit nitrogen-fixing activity under the same conditions tested in the present study (data not shown). The nitrogen-fixing activities and diazotrophic growth of these novel isolates were observed at 70°C. Prior to the present study, the maximum temperature observed for active nitrogen fixation by bacteria was 63.4°C, as demonstrated in cyanobacteria from hot springs (37, 38).

The characteristics of nitrogenase activity in isolates differed from that previously reported for the chemosynthetic microbial communities of Nakabusa hot springs (28). Nitrogenase activities measured in communities under anaerobic conditions were suppressed by more than 95.5% by molybdate (an inhibitor of sulfate reduction and thiosulfate disproportionation [5, 17, 32]). These communities also required H_2_, CO_2_, and sulfate (not thiosulfate) for their nitrogenase activities, suggesting that nitrogenase activity is associated with anaerobic autotrophic sulfate reduction in microbial communities. In contrast, the isolates showed nitrogenase activity with thiosulfate, which was inhibited by molybdate only under anaerobic (thiosulfate disproportionating) conditions. Therefore, the isolates may not be responsible for the previously measured nitrogenase activity (28). We previously analyzed putative *nifH* environmental clone sequences in chemosynthetic microbial communities at Nakabusa hot springs (29). The most abundant operational taxonomic units of *nifH* environmental clone sequences were related to *Aquificae* and the genus *Caldicellulosiruptor* in the phylum *Firmicutes* (29). The NifH sequences of both of the strains isolated in the present study have already been detected in environmental samples from Nakabusa hot springs (Fig. 3). Furthermore, closely related putative *nifH* sequences have been obtained from thermophilic environments in metagenome studies conducted on samples from Yellowstone National Park (USA), and the findings indicated the global distribution of nitrogen-fixing *Hydrogenobacter* in thermal environments (7, 8, 22, 36).

In addition to *nifH* genes, 16S rRNA gene amplicon sequences closely related to those of strain 1–6 have been detected in several types of chemosynthetic microbial mats and streamers in Nakabusa (29). However, the relative abundance of the 16S rRNA gene sequence of strain 1–6 in these environmental samples was low, representing only 0.2–0.6% of the total reads in the microbial mat and streamer samples. The 16S rRNA gene sequence of strain 2–18 was not detected in amplicon analyses; however, the presence of strain 2–18 in the communities in Nakabusa was shown by the detection of its putative *nifH* gene as described above (the relative abundance of the 16S rRNA gene sequence was <0.05%) (Fig. 3). The detection of both strains in environmental samples confirmed the presence of these bacteria in their natural habitat. One possible reason for the differences in the abundance of these two strains is their O_2_ preference. Strain 1–6 preferably grew at 10% vol. O_2_ atmosphere, while strain 2–18 preferred lower O_2_ concentrations of approx. 5% vol., being inhibited by higher concentrations (Fig. 6).

The effects of molybdate on nitrogen fixation were not observed in the presence of both H_2_ and thiosulfate at 5% vol. O_2_ (Fig. 7a and b). These results indicated that the inhibitory effects of molybdate were not directly related to nitrogenase. The decrease that occurred in nitrogenase activity following the addition of molybdate suggested that the energy acquisition process for nitrogen fixation was inhibited. Molybdate is known to inhibit sulfate reduction as well as thiosulfate disproportionation (5, 17). It interferes with the initial step in sulfate reduction, the formation of adenosine-5-phosphosulfate (APS), and the reverse reaction in disproportionation (5, 32). The inhibitory effects of molybdate on the nitrogenase activities of strains 1–6 and 2–18 in the absence of H_2_ and O_2_ indicates the ability for thiosulfate disproportionation in these *Aquificae* isolates, similar to previously reported molybdate inhibition in thiosulfate-disproportionating *Deltaproteobacteria* (5, 17). In the presence of H_2_ and O_2_ with the addition of molybdate, these bacterial strains harness energy for nitrogen fixation by aerobic hydrogen oxidation (Fig. 7a). Strains 1–6 and 2–18 exhibited the ability to grow under hydrogen-oxidizing conditions, as observed for other bacterial species in the phylum *Aquificae* (10). Future studies will elucidate thiosulfate metabolism in these isolates underlying the observed molybdate inhibition.

As shown in our previous study (28), the anaerobic nitrogenase activities of chemosynthetic microbial communities in Nakabusa depended on H_2_, CO_2_, and sulfate, and were markedly inhibited by molybdate, which was interpreted as an indication for the sulfate-reducing chemoautotrophic metabolism of the dominating diazotrophs. Although molybdate-sensitive nitrogenase activity was observed for the new isolates, the metabolic basis of inhibition appears to differ from previous findings obtained in environmental communities. Sensitivity to molybdate in the isolates was dependent on the absence of H_2_ and O_2_, whereas H_2_ production occurred in environmental communities during incubations (data not shown). The presence of diazotrophic and autotrophic sulfate-reducing members in the thermophilic communities at Nakabusa hot springs cannot be excluded based on the data obtained, and further studies are needed to answer this question.

Nitrogen fixation in *Hydrogenobacter* strains appears to require semi-aerobic conditions with lower O_2_ concentrations than atmospheric O_2_ concentrations, in addition to reduced sulfur compounds and/or hydrogen as electron sources. Therefore, microbial mats and streamers appear to be appropriate environments for nitrogen fixation, *i.e.*, the low O_2_ concentration achieved by O_2_ consumption through aerobic respiration, anaerobic sulfide production, sulfide supply from hot spring water, elemental sulfur production by abiotic or biotic processes, and hydrogen production by fermentative bacteria (28, 29, 44). *Hydrogenobacter* species have been shown to widely populate neutral to alkaline environments at a temperature range higher than 70°C, at which nitrogen compounds are limited, and some may be capable of fixing and supplying nitrogen in these communities (3, 7, 8, 22, 29, 41).
